# Quantitative Profiling of *Drosophila melanogaster Dscam1* Isoforms Reveals No Changes in Splicing after Bacterial Exposure

**DOI:** 10.1371/journal.pone.0108660

**Published:** 2014-10-13

**Authors:** Sophie A. O. Armitage, Wei Sun, Xintian You, Joachim Kurtz, Dietmar Schmucker, Wei Chen

**Affiliations:** 1 Institute for Evolution and Biodiversity, University of Münster, Münster, Germany; 2 Laboratory for Novel Sequencing Technology, Functional and Medical Genomics, Berlin Institute for Medical Systems Biology, Max-Delbrück-Centrum für Molekulare Medizin, Berlin, Germany; 3 Laboratory of Neuronal Wiring, Vesalius Research Center, VIB, Department of Oncology, University Leuven, Leuven, Belgium; Ecole Normale Supérieur de Lyon, France

## Abstract

The hypervariable *Dscam1* (Down syndrome cell adhesion molecule 1) gene can produce thousands of different ectodomain isoforms via mutually exclusive alternative splicing. *Dscam1* appears to be involved in the immune response of some insects and crustaceans. It has been proposed that the diverse isoforms may be involved in the recognition of, or the defence against, diverse parasite epitopes, although evidence to support this is sparse. A prediction that can be generated from this hypothesis is that the gene expression of specific exons and/or isoforms is influenced by exposure to an immune elicitor. To test this hypothesis, we for the first time, use a long read RNA sequencing method to directly investigate the *Dscam1* splicing pattern after exposing adult *Drosophila melanogaster* and a S2 cell line to live *Escherichia coli*. After bacterial exposure both models showed increased expression of immune-related genes, indicating that the immune system had been activated. However there were no changes in total *Dscam1* mRNA expression. RNA sequencing further showed that there were no significant changes in individual exon expression and no changes in isoform splicing patterns in response to bacterial exposure. Therefore our studies do not support a change of *D. melanogaster Dscam1* isoform diversity in response to live *E. coli*. Nevertheless, in future this approach could be used to identify potentially immune-related *Dscam1* splicing regulation in other host species or in response to other pathogens.

## Introduction

The *Dscam1* (Down syndrome cell adhesion molecule 1) gene encodes for an unprecedented number of mRNA isoforms in both insects and crustaceans. The gene has been best characterised in *Drosophila melanogaster*, where in addition to 20 constitutively expressed exons, there are 95 variable exons spread across 4 exon clusters. Twelve variable exons are found in exon cluster 4, 48 in cluster 6, 33 in cluster 9, and 2 in cluster 17 [1]. As a result of mutually exclusive alternative splicing, each isoform contains only one of the variable exons from each cluster, which for the extracellular exons clusters (4, 6 and 9), means a theoretical diversity of 19,008 combinations. For clarification we note that the *Dscam1* gene in *D. melanogaster* and its pancrustacean orthologs have been variously named (e.g., *Dscam* [1], [2], *Dscam-hv* [3], [4], *AgDscam* [5]), but we use its most recent identity, i.e., *Dscam1* [6], [7], for all species.

Such isoform diversity has been shown to be essential for cell recognition in the nervous system (e.g., [7]–[14]; for reviews see [6], [15], [16]). For example *Dscam1* regulates self-avoidance, i.e., where neurites from the same cell actively avoid each other, ensuring that they grow into different territories. In this way *Dscam1* can be envisioned as a molecular surface code enabling neurite self-nonself discrimination [6]. Each individual neuron is thought to express a restricted set of possible isoforms [17], [18] that are different to the isoforms expressed by neighbouring cells [6]. When sister neurites express identical (or highly related) isoforms, it leads to homophilic recognition between the two neurites and therefore repulsion [12].

*Dscam1* is also expressed in haemocytes and the fat body [19], both of which have essential immune functions [20], and there is accumulating evidence that *Dscam1* plays a role in insect and crustacean immunity [5], [19], [21], [22] (reviewed in [23]). Within the context of the immune system it has been proposed that *Dscam1* may act as a pattern recognition receptor, meaning that the isoform diversity allows for the recognition of diverse parasite epitopes (e.g., [5], [24]). Considering receptor function and specificity in known immune-defence pathways, particularly in vertebrate adaptive immunity, one might expect that the expression of alternatively spliced variants would differ according to the encountered parasite. Some evidence exists to support this hypothesis: Dong et al [5] exposed a mosquito-derived cell line, Sua5B, to diverse heat-inactivated bacteria (*Escherichia coli*, *Pseudomona veronii* and *Staphylococcus auerus*), bacterial cell wall components (lipopolysaccharide (LPS) and peptidoglycan (PG)) and a fungal parasite (*Beauveria bassiana*), and 12 or 18 hours later they examined the expression of individual exons within cluster 4 using real-time quantitative PCR (RTqPCR). They found, as predicted, that expression of the alternative exons varied according to the infecting parasite, furthermore the expression pattern at 12 hours after exposure to *E. coli* and *S. aureus* significantly correlated with the pattern for the respective bacterium at 18 hours [5]. Adult mosquitoes, *Anopheles gambiae*, showed similar exon 4 splicing patterns as the Sua5B cells after exposure to *E. coli*, but not after exposure to *S. aureus*, and splicing patterns in the midgut after *Plasmodium* infection showed different patterns again [5].

A number of other studies have addressed a similar question by PCR amplification of cDNA from the alternatively spliced regions of *Dscam1* followed by cloning and then sequencing; broadly speaking they have found groups of exons within the variable exon clusters that showed parasite dependent changes in expression compared to uninfected controls. More specifically, exposure of the whiteleg shrimp (*Litopenaeus vannamei*) to white spot syndrome virus resulted in some isoforms from each of the variable regions being preferentially associated with different disease states [25]; Smith et al [26] exposed *A. gambiae* to the protozoan *Plasmodium falciparum* and then grouped sequenced exons according to genetic distance and found that one group of exon 4 and one of exon 6 were underrepresented in control mosquitoes, and that there was an increase in the combined diversity of expressed exons 4 and 6 in mosquitoes exposed to *P. falciparum*. Furthermore, Watthanasurorot et al [22] injected the signal crayfish (*Pacifastacus leniusculus*) with *E. coli* and *S. aureus* and did a multiple sequence alignment of the sequenced cDNA and found that the isoform sequences tended to cluster according to the bacterial exposure treatment; similarly, exposing the whiteleg shrimp to the bacterium *Vibrio harveyi* resulted in the clustering of a subclade with more related isoforms in the exposed group [27]. Therefore there appears to be no increased representation of one particular exon from each variable cluster; the studies instead show that the expression of a few *Dscam1* alternatively spliced exons can potentially be influenced by parasite exposure.

Given the tens of *Dscam1* exon variants within each alternatively spliced cluster, and the potentially thousands of isoforms that can result from the splicing together of these exons, RNA deep sequencing would be a particularly suitable methodology to further our understanding of splicing patterns after parasite exposure. The long read lengths that are made possible by using Pacific Biosciences technology give the added advantage of providing sequences that are long enough to illuminate whole isoform sequences, i.e., combinations of specific exon 4, 6 and 9 variants. Such methods have been able to detect up to 18,496 of the possible isoform combinations [2], but have not, to date, been used to address the immune-related aspect of *Dscam1*’s function.

Important pioneering work on insect immunity has been performed on *D. melanogaster* (reviewed in [20]) and evidence exists to suggest that *Dscam1* plays a role in the immune system of *D. melanogaster* as well as a cell line derived from this species [19]. In this study we used adult *D. melanogaster* and the *Drosophila* Schneider 2 (S2) cell line to first test whether *Dscam1* shows increased general expression after bacterial exposure, and second whether exons in alternatively spliced exon clusters 4, 6 and 9 show bias in expression after bacterial exposure. We exposed adult flies to *E. coli* for 18 hours, and the S2 cell line for 12 and 18 hours because these time frames were previously used by Dong *et al*. to examine acute phase *Dscam1* splicing patterns in *A. gambiae* [5]. To investigate *Dscam1* splicing patterns we used deep sequencing based methods [2] to quantitatively profile *Dscam1* mRNA; this methodology has the advantage of allowing for a much higher coverage of each exon than RTqPCR or cloning and sequencing methods, and it also allows one to eliminate potential biases from cloning steps. Although we found that the immune system was activated in response to *E. coli*, demonstrated by increased antimicrobial peptide (AMP) gene expression, we did not detect any significant overall increase in *Dscam1* expression. Furthermore, through RNA deep sequencing, we did not detect any differences in the splicing pattern in the *E. coli* exposed treatments compared to the control treatments.

## Materials and Methods

### Fly strain, cell line and bacteria

For the *in vivo* experiment we used a wild type *D. melanogaster* (FlyBase ID: FBst0025174) stock, which is a homozygous isogenic strain originating from Raleigh, USA that has been sequenced and phenotypically characterised [28]. The flies were maintained at 25°C, 70% humidity, on a 12∶12 hour light:dark cycle, with non-overlapping generations, and kept on a diet containing 17.9 g brewers yeast, 35.7 g malt extract, 71.4 g corn flour, 10 g soy flour, 4 g sugar beet syrup, 10.7 g agar, 1.4 g nipagin, 4 g propionic acid and 1 L water. For the *in vitro* experiment we used *Drosophila* Schneider 2 (S2) cells (Invitrogen). The S2 cells were derived from a primary culture of late embryonic stage *D. melanogaster* embryos [29], and are appropriate to use in an immune context because they show haemocyte-like characteristics in terms of gene expression and cellular behaviour, such as phagocytosis [30]–[32]. The S2 cells were maintained at 25°C in serum free medium (Sf900; Gibco) containing 2 mM L-glutamine (hereafter termed culturing medium), and split twice weekly by diluting the cells 1∶5 in fresh medium. For bacterial exposure of the flies and S2 cells we used *Escherichia coli* (K12 wildtype; German Collection of Microorganisms and Cell Cultures; DSM No: 498). As far as was possible, the experiments were performed blind with respect to treatment, and treatment orders were randomised within each experimental block.

### Adult fly exposure to *E. coli* and RNA extraction

To raise adult flies, we collected eggs from females that had been allowed to oviposit on 1.5% agar plates containing 1% vinegar and spread with a paste of live yeast. Before hatching, the eggs were washed from the plates with PBS and added to fresh food vials to achieve a density of around 140 eggs per vial (methods after [33]). Upon imaginal eclosion, mixed sex groups of flies (50 females and 50 males) were placed into fresh food vials, and maintained there until a minimum of six days post eclosion. At this point, the flies were briefly anaesthetised using CO_2_ and sorted on ice into single sex groups of six flies until the bacterial challenge. Prior to inoculation *E. coli* was cultured for eight hours in lysogeny broth (LB) at 30°C, 200 rpm, then centrifuged (2880 RCF) and washed twice in *Drosophila* Ringer’s solution [34]. The concentration was adjusted to 1×10^10^ bacterial cells per mL: *E. coli* is non-lethal to the flies and preliminary experiments showed that this concentration of bacteria resulted in 100% survival 72 hours after pricking (n = 48). On each of two consecutive days we produced three treatment groups for RTqPCR, two of which were used for RNA sequencing. One group received a septic wound whereby the flies were anaesthetised on ice and the bacteria were introduced into the haemocoel by piercing the lateral side of the thorax with a fine pin (diameter 0.05 mm) that had been dipped into the *E. coli* solution (n = 24 females and 24 males from the two days; hereafter named *E. coli* exposure group). The second group was a control, i.e., flies received only ice anaesthesia (n = 24 females and 24 males from the two days; hereafter named control group). The third group were pierced with a fine pin dipped in *Drosophila* Ringer’s solution (hereafter named wounded); they were produced for RTqPCR only, because wounding itself can produce an immune response and result in the expression of the AMPs that we used as positive controls (e.g., [35], [36]; see below for details of positive control gene expression); therefore they were used to examine whether the immune response after *E. coli* exposure was greater than wounding alone, relative to the control group. Six flies were prepared at a time, in a random order with respect to treatment. After treatment, the flies were kept in single sex groups of six flies for eighteen hours; after this time we ice-anaesthetised the flies and carefully removed the head (to remove the potential ‘contaminating’ *Dscam1* expression from the brain) and froze the rest of the body in liquid nitrogen and stored it at −80°C. Before RNA extraction we pooled the bodies into two biological replicates for each treatment group, each containing ten females and ten males. One mL of TRIzol reagent (Ambion, U.S.A) was added to the 20 frozen homogenised bodies, and RNA extraction was performed according to the FlyChip protocol: ‘Standard protocol for the extraction of total RNA from *Drosophila melanogaster*’ (www.flychip.org.uk/standard_extraction.php). After extraction the RNA pellet was resuspended in 20 µl of nuclease free water; the integrity and concentration of the RNA were estimated using an Agilent 2100 Bioanalyzer and a NanoPhotometer Pearl. One µL of each sample was frozen separately at −80°C for RTqPCR (n = 2 per treatment group), and the remainder of the samples were pooled for each treatment (n = 1 per treatment group) and stored at −80°C until RNA sequencing.

### S2 cell exposure to *E. coli* and RNA extraction

The S2 cells were brought up to 5.9×10^6^ cells/mL and added in 850 µl aliquots (replicates) to 12-well plates. To the 18 hour *E. coli* exposure group and its corresponding control we added 50 µl of Penicillin-Streptomycin (final concentration 10 µg/mL): our preliminary experiments showed that antibiotics were necessary to ensure that the S2 cells had a high survival after this exposure period. At the same time, we added 50 µl culturing medium to the 12 hour exposure group. The cells were incubated overnight under standard culture conditions. The inoculation *E. coli* was produced in the same way as for the fly infections, except that it was washed twice in LB and then once in culturing medium. Prior to inoculation, we estimated the concentration of the S2 cells from one of the replicates and adjusted the *E. coli* concentration such that for the 12 hour exposure the starting ratio of S2: *E. coli* would be 1000∶1, and for the 18 hour exposure the ratio would be 100∶1. We added 100 µl of the corresponding *E. coli* or 100 µl culturing medium to the controls. The plates were gently swirled and centrifuged for 5 minutes, then returned to standard culturing conditions. Twelve or eighteen hours later the cells were thoroughly resuspended, the contents of each well added to one 1.5 mL reaction tube, and centrifuged at 300 RCF for 5 minutes [37]. The supernatant was replaced with 1 mL TriZOL reagent, and a 23 G syringe was used to lyse the cells. The cells were frozen immediately in liquid nitrogen and transferred to −80°C until the extraction of RNA. Three replicates from each of the four treatment groups were later used for RTqPCR, and three replicates from each of the four treatments were pooled and later used for sequencing. At both sampling times we estimated the S2 cell viability from control and bacterially-exposed replicates (Survival for 12 hr control: 100%; 12 hr *E. coli*: 98.6%; 18 hr control: 99.1%; 18 hr *E. coli*: 97.2±0.3% (±1 s.e.)). RNA extractions for RTqPCR were performed similarly to the protocol described above for the flies, except that after separation of the aqueous phase the samples were further processed with the SV Total RNA Isolation System (Promega). RNA for sequencing was isolated using TriZOL according to the manufacturer’s instructions (Life Technologies). The integrity and concentration of the RNA were estimated using an Agilent 2100 Bioanalyzer and a NanoPhotometer Pearl.

### Reverse Transcription and Real Time quantitative PCR

We used RTqPCR to test whether total *Dscam1* expression was altered in the flies or the S2 cells after bacterial exposure and to test whether an immune response had been elicited. To reverse transcribe the fly and S2 cell RNA, SuperScript III (Invitrogen) was used according to the manufacturer’s instructions using random hexamer primers and 500 ng RNA as a template. The resulting cDNA was diluted 1∶10 (fly samples) or 1∶20 (S2 cell samples) and used for RTqPCR, with a primer pair designed from two constitutive *Dscam1* exons, and primers from the antimicrobial peptide genes *Diptericin* and *Drosomycin* as positive controls [38] (see Table 1 for all primer sequences). The reference gene was *rpL13a* (Table 1), and where possible, one primer for each gene spanned an exon-exon boundary. The qPCR was performed in a 384-well plate format, with a total reaction volume of 10 µl in each well. From each cDNA sample two technical replicate qPCR reactions were performed using the Kapa SYBR Fast qPCR Mastermix according to the manufacturers instructions. The reaction was run on a LightCycler480 (Peqlab Ltd) using the following protocol: 95°C for 3 min, followed by 40 cycles of annealing and amplification at 60°C for one min. As a final step the products were heated up to 95°C with continuous fluorescence measurements to obtain the melting curves and subsequently cooled to 40°C. The crossing point (Cp) values (Table S1) were calculated using the second derivative maximum method [39] with the LightCycler480 software and the average Cp values from each of two technical replicates were used for analyses. The data were analysed using REST 2009 (relative expression software tool; [40]), which allows one to compare the gene expression of two groups at a time: the *E. coli* exposed flies or S2 cells were tested against the control flies or S2 cells, and in a separate analysis the wounded flies were tested against the control flies. REST calculates the relative fold expression differences by using the expression of a reference gene (*rpl13a*) to normalise the expression levels of the target genes (*Dscam1, Diptericin* and *Drosomycin*), whilst taking the reaction efficiency (E) of the PCR into account [40], [41]. REST performs a pair wise fixed reallocation randomisation test to examine whether there are significant differences between the two groups. We allowed 2000 random reallocations of the observed Cp values to the two groups being tested; REST notes the expression ratio change for each reallocation, and the proportion of these effects gives the p-value assuming a 2-sided test [40]. In our figures we present the means and standard errors as calculated according to the REST software, i.e., the results of the 2000 random reallocations.

**Table 1:** Sequences of all used primers.

Gene	FlyBase ID	Efficiency (*E*)	5′–3′ primer sequence	Use	Primerorigin
*Diptericin*	FBgn0004240	1.979	F: GCTGCGCAATCGCTTCTACT	RTqPCR	[38]
			R: TGGTGGAGTGGGCTTCATG		
*Drosomycin*	FBgn0010381	1.945	F: CTGCCTGTCCGGAAGATACAA	RTqPCR	[56]
			R: TCCCTCCTCCTTGCACACA		
*Dscam1* exons10 & 10/11	FBgn0033159	2.0	F: TAAGGCCTTCGCCCAGGGATCC	RTqPCR	Unpublished data
			R: TCTCCGGGGGTGTCGCCAACT		
*rpL13a*	FBgn0037351	1.98	F: AAGGCAGTCCGAGGCATGATCCC	RTqPCR	Unpublished data
			R: CGACGCTTGTCGTAGGGCGA		
*Dscam1* exon 11			GTCGCTCTTCTTTAGATCCTTGTAC	RT	[2]
*Dscam1* exons3 & 10*			F: AGGGATACCATTATCTCCCGGGACGTCCATGTGC	Sequencing	[2]
			R: GTGGATACCTTATCGGTGGGCTCGAGGATCCA		

*Sequences include 7 bp barcode.

### Reverse transcription and PacBio sequencing for quantifying *Dscam1* alternatively spliced exons and isoform abundance

PacBio (Pacific Biosciences) RS system sequencing for quantifying *Dscam1* isoform abundance was performed as described previously [2]. RT was performed on 5 µg of total RNA from either a fly sample or S2 cells, with a primer annealed to constitutive exon 11 (Table 1) and using SuperScript III with a reaction volume of 20 µl. The first round PCR followed using 2 µl of RT product as a template in 25 µl of Advantage 2 PCR system. The PCR primers were targeted at constitutive exons 3 and 10, with 7 base pair barcode sequences attached at the 5′ ends (Table 1). The PCR was run as follows: 2 min at 95°C, followed by 22 cycles of 30 sec at 95°C, and 2.5 min at 72°C, and a final elongation of 10 min at 72°C. The PCR products were purified using Agencourt AMPure XP system (Beckman Coulter). After the measurement of concentrations and fragment size with the Qubit system and Agilent 2100 Bioanalyzer, purified PCR products were directly sequenced using PacBio RS system according to the manufacturer’s instruction. Circular consensus reads obtained from PacBio sequencing were aligned to *Dscam1* reference exons (http://www.ncbi.nlm.nih.gov/nucleotide/AF260530?tool=FlyBase) using BLAT (parameters: -tileSize = 8 -stepSize = 5 -oneOff = 1 -minScore = 20 -minIdentity = 70). We retained the sequences if, and only if, the identity of exon 4, exon 6 and exon 9 could all be unambiguously revealed [2]. We tested whether there was a statistical difference in expression between the control and *E. coli* exposed treatments for exon 4, 6 and 9 variants by using DESeq [42] within the R statistical package [43]; p-values were corrected for multiple testing using the Benjamini & Hochberg [44] method.

## Results and Discussion

### *E. coli* exposure activated an immune response

Upon the introduction of bacteria or fungi into the body cavity of insects there is a so-called systemic immune response, whereby two evolutionarily conserved signalling pathways, Imd and Toll, lead to the secretion of AMPs into the haemolymph to destroy the infecting microorganisms [20], [45]. To demonstrate that we had activated the fly and S2 immune response after exposing them to *E. coli*, we took advantage of the fact that activation of the Imd and Toll pathways can be monitored by measuring AMP gene expression after bacterial infection [38]. Therefore we measured the expression of two *D. melanogaster* AMP genes, *Diptericin* [46] and *Drosomycin* [47]. Gene expression of *Diptericin* is regulated by Imd pathway induction, and is produced by the fat body after activation by diaminopimelic acid-type peptidoglycan (DAP) found in most Gram-negative bacteria [36], [38], therefore we expected upregulation in the flies after *E. coli* exposure. *Drosomycin* is mainly regulated by the Toll pathway in response to fungi but can also be induced in response to Gram-negative bacteria [48], [49], therefore we predicted no, or a small, increase in *Drosomycin* gene expression after exposure (e.g., [35], [49]). The extent to which AMP expression increases after bacterial exposure and the time point of peak AMP expression vary according to the bacterial species examined, but with the time frame and bacteria species that we used it would be reasonable to expect at least an increase in *Diptericin* expression in the flies (e.g., [35], [48]). AMP secretion by S2 cells, particularly in response to live extracellular bacteria, has been examined to a lesser extent than in the flies. However there is clear evidence that S2 cells transcribe AMPs, including *Diptericin* and *Drosomycin*, in response to bacterially-derived immune stimulants and bacteria (e.g., [50]–[52]), and that both of these AMPs are expressed after exposure to heat-killed *E. coli* [53], [54]. Therefore we also predicted increased expression of our two chosen AMPs in the S2 cells after exposure to live *E. coli*.

**Figure 1:**
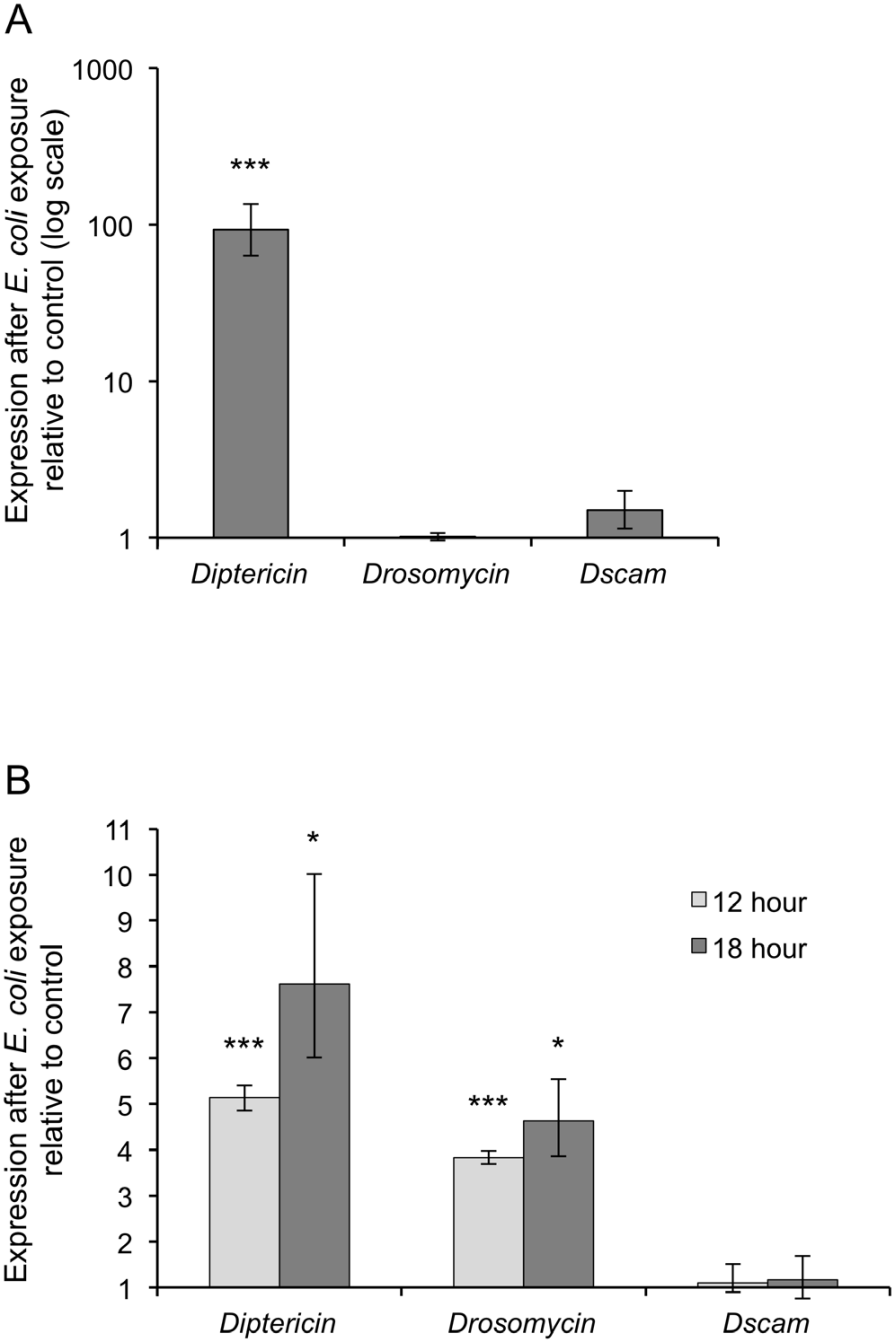
Fly and S2 cell expression profiles of *Diptericin*, *Drosomycin* and *Dscam1* after *Escherichia coli* exposure. A | At 18 hours after pricking with *E. coli* flies showed a significant increase in *Diptericin* but no significant change in *Drosomycin* or *Dscam1* expression when compared to the control flies. n = 2 biological replicates for *E. coli* and control groups, each replicate containing 20 flies. B | Both 12 and 18 hours after the addition of *E. coli*, S2 cells showed a significant increase in antimicrobial peptide (*Diptericin* and *Drosomycin*) expression, but no significant change in *Dscam1* expression when compared to the control cells. n = 3 biological replicates for *E. coli* and control groups. The expression of the reference gene *rpl13a* was used to normalise the expression of the target genes. Means and standard errors were calculated according to [41] using the REST software [40]; Statistically significant differences between *E. coli* exposed groups and the control groups are indicated by * for p<0.05 and *** for p<0.001.

Our results showed that both the flies and S2 cells upregulated expression of antimicrobial peptides after *E. coli* exposure (Figure 1), indicating that the bacteria had successfully induced an immune response. The flies significantly increased expression of *Diptericin*, but not *Drosomycin*, in response to *E. coli* (Figure 1A). As expected, wounding alone also resulted in significantly increased *Diptericin* gene expression relative to the control group (e.g., [35], [36]), although this was lower than *Diptericin* expression after *E. coli* treatment relative to the control group (Figure S1). In contrast to the flies, the S2 cells showed a significant increase in expression of both AMPs at 12 and 18 hours, however this was to a lower degree compared to controls in comparison to the flies (Figure 1B), and one should bear in mind that this might indicate a lower level of immune responsiveness compared to the flies, and furthermore that antibiotics were required to enhance the survival of the cells in the 18 hour exposure treatment. The degree of AMP upregulation that we found was in the range of some S2 cell studies (e.g., [51], [52]) but other studies have shown higher upregulation in response to other immune stimulants (e.g., [50]).

### *E. coli* exposure did not lead to an increase in *Dscam1* gene expression

Despite activation of the immune system as evidenced by increased AMP expression, we did not find an overall increase in *Dscam1* expression either 12 or 18 hours after *E. coli* exposure of S2 cells, and 18 hours after exposure of adult *D. melanogaster* (Figure 1). One caveat that should be borne in mind is that we had low sample sizes (two biological replicates for each treatment each containing 20 flies) meaning that our power to detect a small effect size was low. Therefore a more conservative interpretation is that our data is consistent with the idea that there is no substantial increase in *Dscam1* expression after *E. coli* exposure at these time points after infection. In the cases where other studies have examined overall *Dscam1* expression after *E. coli* exposure, varied results have been found (for a review see [23]), even within the same host exposed to different pathogens: for example, Watthanasurorot et al [22] found increased *Dscam1* expression in the signal crayfish after the injection of *E. coli*, LPS and *S. aureus*, but not in response to PG or white spot syndrome virus. The increase in expression was found 6, 12 and 24 hours after injection [22], therefore within the same time frame as our experiments. Another crustacean species, the whiteleg shrimp, also showed upregulation of *Dscam1* in response to some parasites but not others [27], [55], and in response to eukaryotic parasite infection, the honeybee upregulated *Dscam1* expression at certain time points but not others.

### *E. coli* exposure did not lead to a change in the pattern of alternatively spliced *Dscam1* exons or isoforms

**Figure 2:**
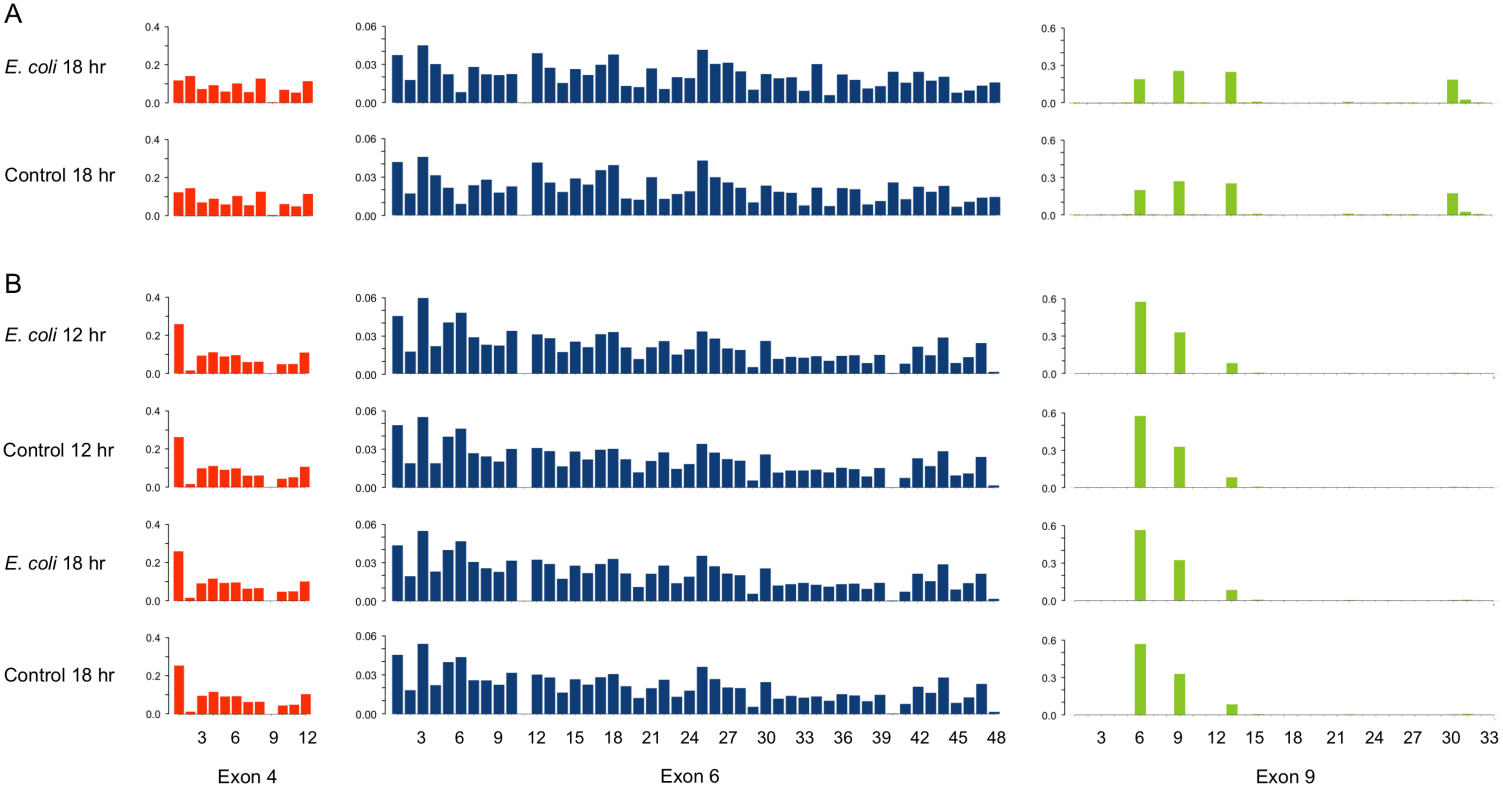
Fly and S2 cell relative expression of *Dscam1* variable exons 4, 6 and 9. A | Relative expression of *D. melanogaster Dscam1* exon variants 18 hours after *Escherichia coli* exposure and the corresponding control. B | Relative expression of S2 cell *Dscam1* exon variants 12 and 18 hours after *E. coli* exposure and the corresponding controls. The y-axes indicate the proportion expression of each exon variant for each cluster.

We tested whether exposure to *E. coli* resulted in a change in the pattern of alternatively spliced exons; this bacteria was chosen because Dong et al [5] found that adult *A. gambiae* and a mosquito cell line showed exon 4 expression bias after *E. coli* exposure. However, unlike Dong et al [5] we used live *E. coli*, because *D. melanogaster Dscam1* has been found to bind better to live rather than heat-inactivated bacteria [19]. To maximise our chances of detecting splicing variant differences if they exist, in the adult fly exposure we used a full untreated control rather than a wounded control group. After exposure to *E. coli*, the flies showed no statistically significant differences in exon representation for any of the three clusters when compared to the control group (Figures 2A & S2A; see Table S2 for sequencing read numbers and statistical results), suggesting that *E. coli* exposure does not affect *Dscam1* splicing patterns under the conditions that we tested. Although we removed the heads of the flies, tissues and cell types other than immune-related haemocytes and the fat body, e.g., the thoracic ganglia and reproductive organs, will have been included in our samples and therefore potential changes might have become diluted. However, it is worth noting that despite the potential problem of testing a mix of tissues and cell types, RTqPCR from whole *A. gambiae* showed differential expression of exon 4 splice variants after *E. coli* challenge [16]. Regarding isoform associations, we note that the heterogeneous cell and tissue mix from the fly also result in a high diversity of sequenced isoforms making it difficult to compare the combinations of exons (isoforms) expressed in the two fly treatments, therefore we restrict the isoform analysis to the S2 cell sequences. Future work would benefit from using an organism with a considerably higher haemocyte or fat body mass than *D. melanogaster* so that pure immune cells/tissues could be RNA sequenced. Except for exon 4.9, which was expressed more than an order of magnitude lower than the other exon 4 variants, and 6.11, which was never expressed and is considered a pseudo-exon [2], most of the exon 4 and 6 cluster variants were sequenced multiple times (Table S2). However, exon 9 expressed a restricted set of 4 variants, as has been described previously particularly for larvae [2] and interestingly also for haemocytes [12].

At both 12 and 18 hours post *E. coli* exposure the S2 cells also showed no differences in exon representation for any of the three alternative exon clusters compared to the controls (Figures 2B & S2B & C; Table S2), corroborating with the results that we found for the flies. Furthermore there was a strong correlation between the number of times each isoform was sequenced in the control and *E. coli* exposed groups (Figure S3), suggestive of no clear bias in the combinations of specific exon 4, 6 and 9 variants after bacterial exposure in the more abundant isoforms. Because we sampled different fly tissues we were not able to make direct comparisons between our fly sequencing results and fly sequencing results from Sun et al [2], however we were able to make across-study comparisons for the S2 cells. When we did this we found that isoform frequencies from all four S2 treatments in this experiment correlated highly with isoform frequencies from Sun et al [2] (Figure S4). We would like to note that this correlation exists despite the fact that this experiment and that from Sun et al [2] were performed under different experimental conditions. Furthermore the splicing patterns that we have found are quite different when one compares the fly and S2 samples (Figure 2): Sun et al also found that S2 cells and fly samples showed different isoform repertoire from one another [2].

Given that we found no difference between *E. coli* exposed and control groups, at first glance our results for the exon 4 cluster may appear to be contrary to results found previously in *A. gambiae* [5], [26] and *P. leniusculus* [22]. However, there are a number of biological reasons other than those already discussed that may help to explain this difference, for example we used a different host species and it is possible that *Dscam1* alternatively spliced exon use after immune stimulation differs across pancrustacean species. Furthermore we used live *E. coli* (*versus* heat inactivated *E. coli* used by Dong et al [5]), and the dose and bacterial strain also differed (e.g., Dong et al [5] used an *E. coli* cloning strain, DH5α). If *Dscam1* splicing responds to dead bacterial cell wall components or to immune-stimulating molecules that might be released after heat-killing, or if our exposure doses were too high or low, for example, then we might not have picked up splicing differences. Furthermore, perhaps *D. melanogaster Dscam1* splicing responds to infections with more naturally infecting micro- or macroparasites, rather than towards *E. coli*, or perhaps it responds after secondary exposure to the same parasite. At present these remain open questions.

To conclude, our results show that *Dscam1* splice variant expression does not differ significantly between control and *E. coli* exposed groups for either adult *D. melanogaster* or S2 cells. These data represent only one of a vast number of possible host-pathogen/parasite interactions. In order to test the general significance, and also the conditions under which, similarly to *A. gambiae*, splicing patterns respond to parasite infections, future studies are needed to test other pathogens and parasites and other potentially more ecologically relevant conditions.

## Supporting Information

Figure S1 **Fly expression profiles of** ***Diptericin*** **,** ***Drosomycin*** **and** ***Dscam1*** **after wounding and** ***Escherichia coli*** **exposure.** At 18 hours after wounding or pricking with *E. coli* there was a significant increase in *Diptericin* expression relative to the control group (means: wounding = 13.5; *E. coli* = 92.8). There was no significant change in *Drosomycin* or *Dscam1* expression. n = 2 biological replicates for *E. coli*, wounded and control groups, each containing 20 flies. Means and standard errors were calculated according to Pfaffl [41] using the REST software [40]. The means for the *E. coli* group are the same as those presented in Figure 1, and are included here for the sake of comparison with the wounded treatment. Means that are significantly different from the control group are indicated by *** for p<0.001.
(TIFF)

Figure S2 **Correlations between the frequencies of** ***Dscam1*** **variable exons sequenced. A** | Correlation between the frequency with which *Dscam1* variable exons were sequenced for the control and *Escherichia coli* exposed flies. **B & C** | Correlations between the frequency with which *Dscam1* variable exons were sequenced for control and *E. coli* exposed S2 cells for the 12 and 18 hour exposures respectively. R-square values for the correlations and the corresponding p-values are in the bottom right corner of each scatterplot.
(TIFF)

Figure S3 **Correlations between S2 cell** ***Dscam1*** **isoform frequencies.** Correlations between the frequency with which *Dscam1* isoforms were sequenced for control and *E. coli* exposed S2 cells for **A** | the 12 hour exposure and **B** | the 18 hour exposure. R-square values for the correlations and the corresponding p-values are in the bottom right corner of each scatterplot.
(TIFF)

Figure S4 **Correlations between S2 cell** ***Dscam1*** **isoform frequencies from this experiment and from Sun et al** [2] **.** Correlations between Sun et al [2] and **A** | S2 *E. coli* 12 hour exposure; **B** | S2 Control 12 hour exposure; **C** *|* S2 *E. coli* 18 hour exposure; **D** *|* S2 Control 18 hour exposure. *Dscam1* isoform frequences from all four S2 treatments in this experiment correlated highly with isoform frequencies of the S2 cell isoform frequencies in Sun et al [2]: R-square values for the correlations and the corresponding p-values are in the top left corner of each scatterplot.
(TIFF)

Table S1 **Mean crossing point (Cp) values from the qPCRs.** The Cp values of the four genes examined for each fly and S2 cell biological replicate. Mean expression differences between the treatment groups as calculated according to Pfaffl [41] using the REST software [40] are also presented, as well as the p-value for whether the expression is significantly different between the treatment groups. The expression differences are are graphed in Figures 1 and S2.
(XLSX)

Table S2 **PacBio RNA sequencing read numbers.** The overall read numbers and the numbers of reads per alternatively spliced exon variant for each treatment. P-values and adjusted p-values for tests of whether there is a statistically significant difference in expression of individual exon variants between the control and *E. coli* exposed treatments are also presented.
(XLSX)
