# Supplementary figures and images for: Quantitative Profiling of Drosophila melanogaster Dscam1 Isoforms Reveals No Changes in Splicing after Bacterial Exposure
Source: PLoS One. 2014 Oct 13;9(10):e108660. doi: 10.1371/journal.pone.0108660 (PMC4195611; doi:10.1371/journal.pone.0108660)

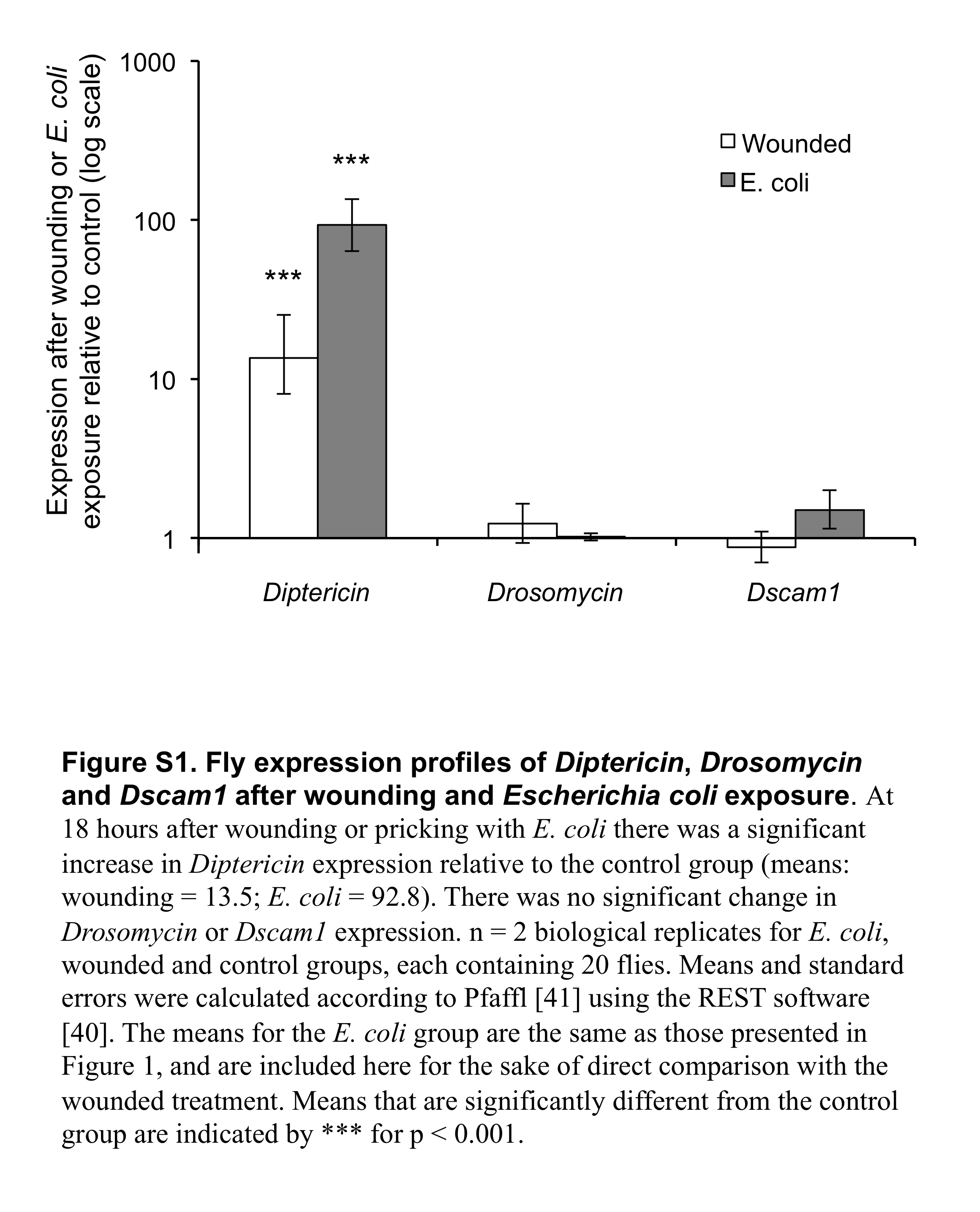

Supplement: Figure S1 — Fly expression profiles of Diptericin , Drosomycin and Dscam1 after wounding and Escherichia coli exposure. At 18 hours after wounding or pricking with E. coli there was a significant increase in Diptericin expression relative to the control group (means: wounding = 13.5; E. coli = 92.8). There was no significant change in Drosomycin or Dscam1 expression. n = 2 biological replicates for E. coli, wounded and control groups, each containing 20 flies. Means and standard errors were calculated according to Pfaffl [41] using the REST software [40]. The means for the E. coli group are the same as those presented in Figure 1, and are included here for the sake of comparison with the wounded treatment. Means that are significantly different from the control group are indicated by *** for p<0.001. (TIFF) [file pone.0108660.s001.tiff]

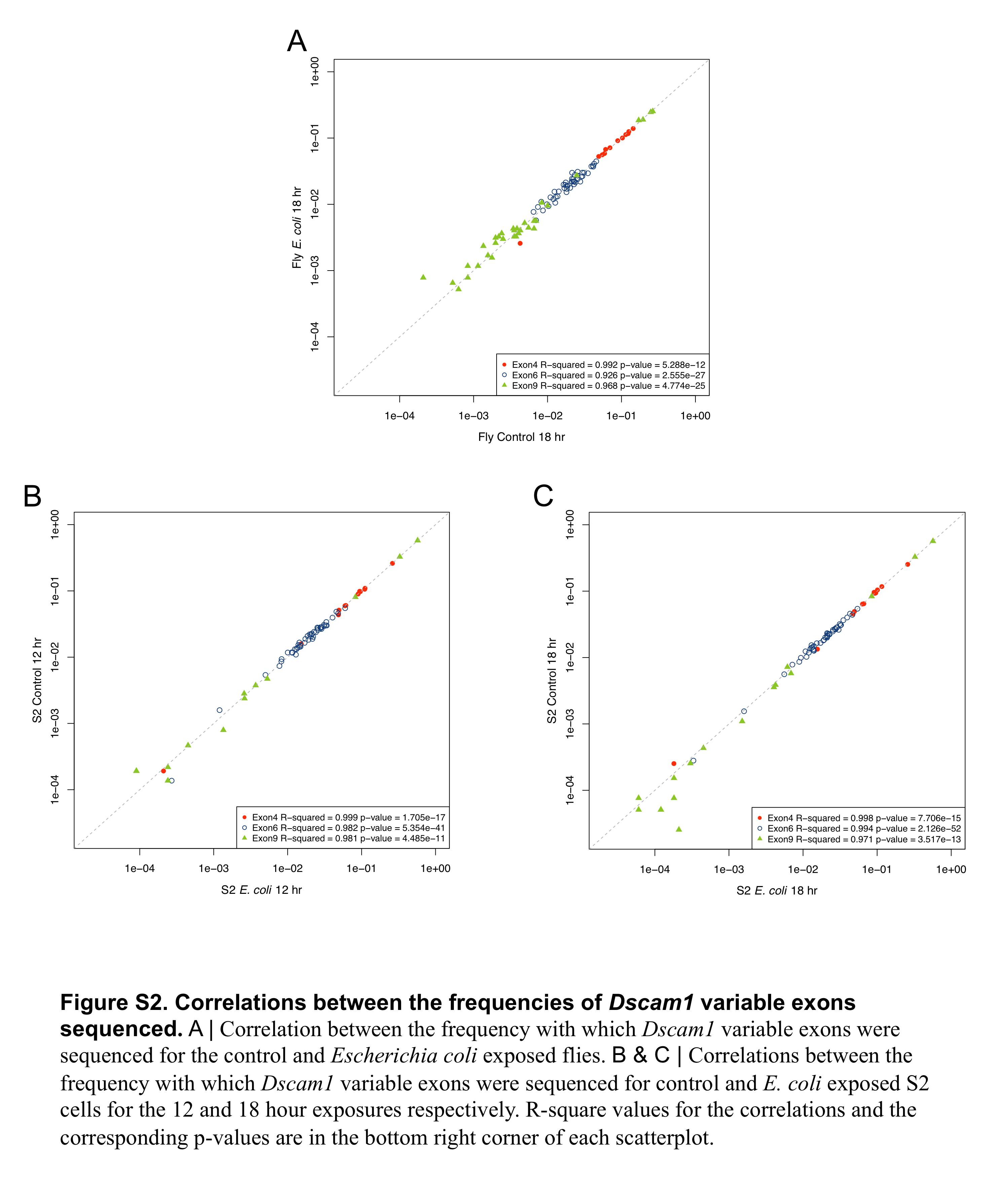

Supplement: Figure S2 — Correlations between the frequencies of Dscam1 variable exons sequenced. A | Correlation between the frequency with which Dscam1 variable exons were sequenced for the control and Escherichia coli exposed flies. B & C | Correlations between the frequency with which Dscam1 variable exons were sequenced for control and E. coli exposed S2 cells for the 12 and 18 hour exposures respectively. R-square values for the correlations and the corresponding p-values are in the bottom right corner of each scatterplot. (TIFF) [file pone.0108660.s002.tiff]

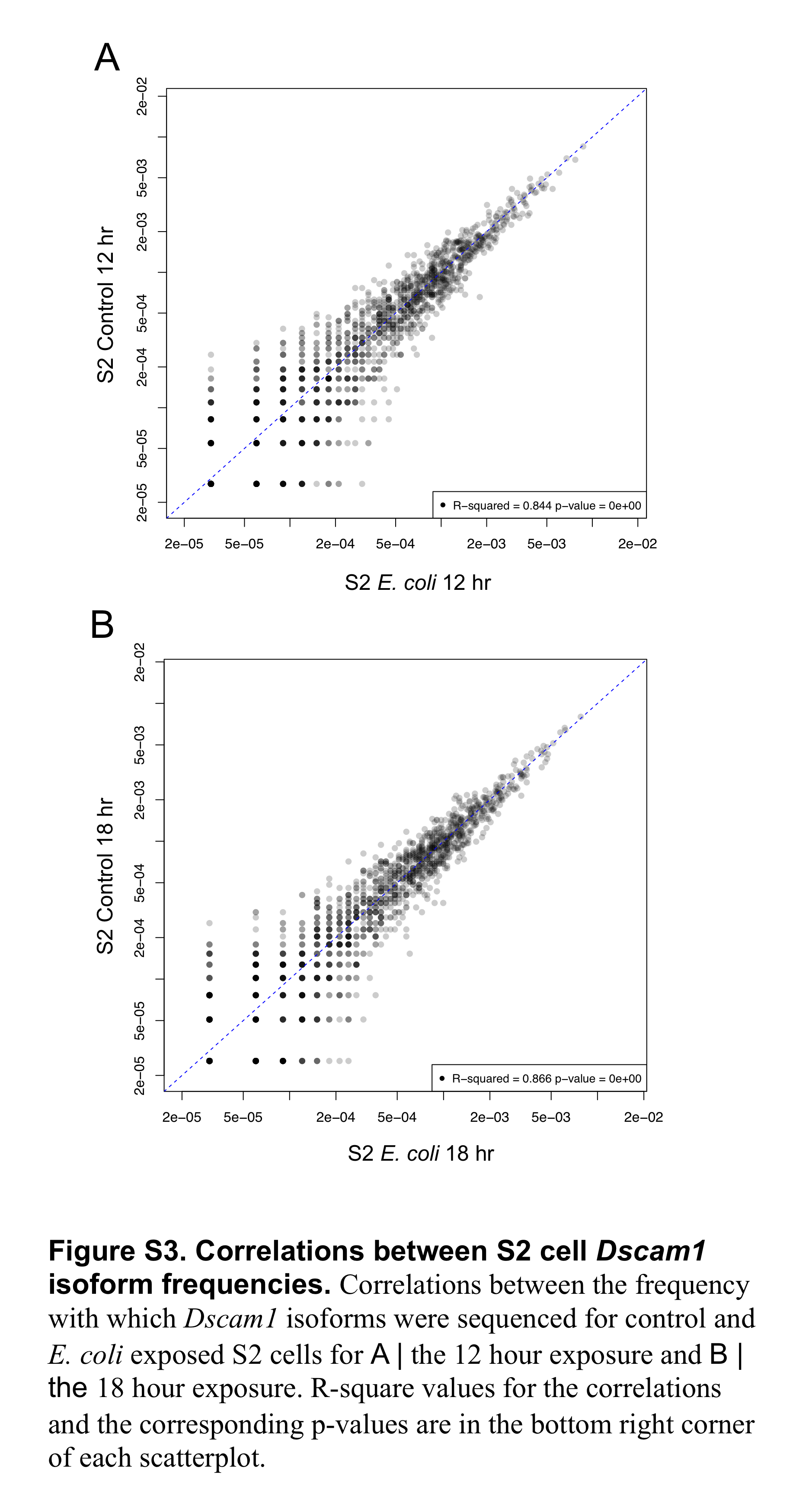

Supplement: Figure S3 — Correlations between S2 cell Dscam1 isoform frequencies. Correlations between the frequency with which Dscam1 isoforms were sequenced for control and E. coli exposed S2 cells for A | the 12 hour exposure and B | the 18 hour exposure. R-square values for the correlations and the corresponding p-values are in the bottom right corner of each scatterplot. (TIFF) [file pone.0108660.s003.tiff]

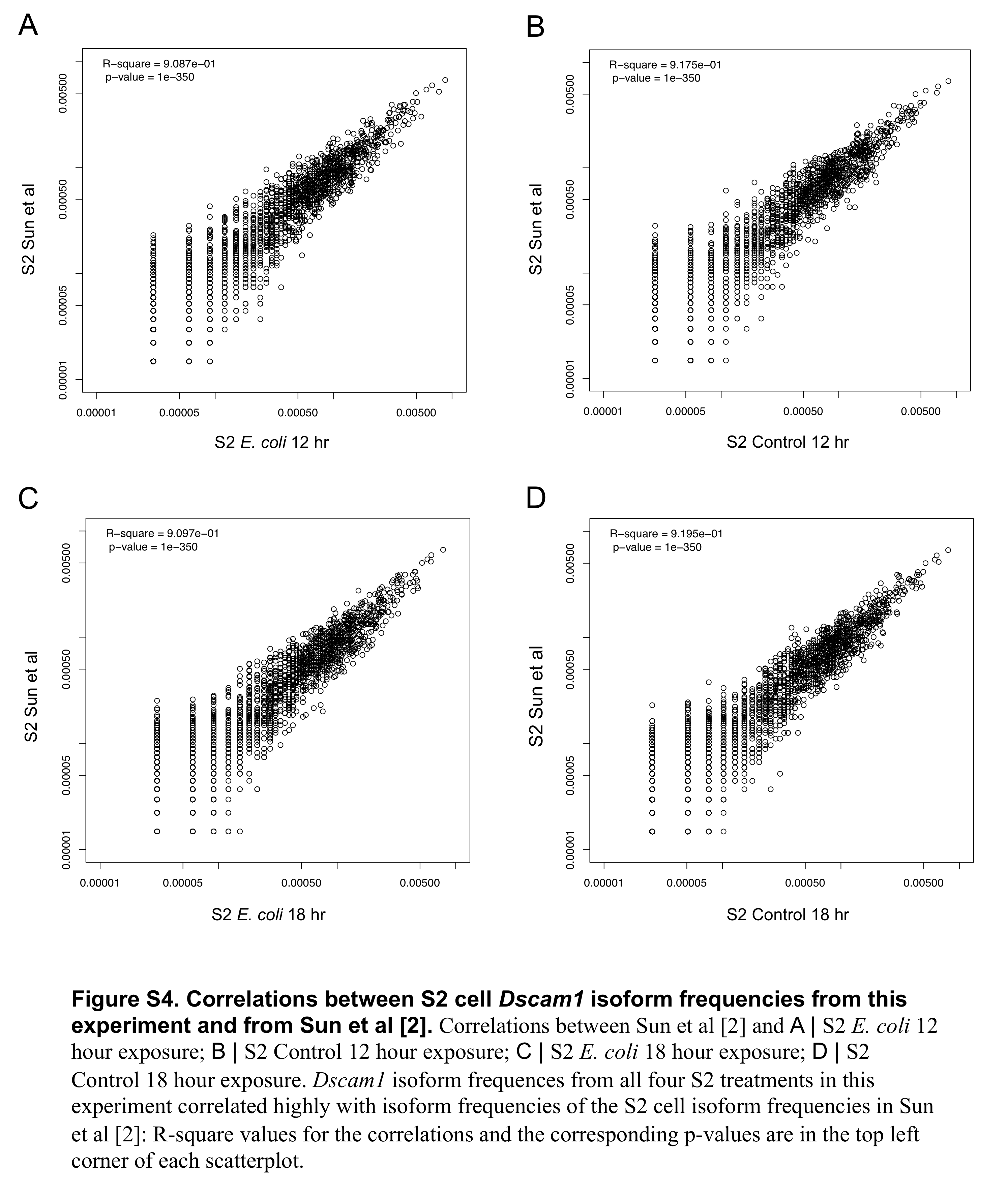

Supplement: Figure S4 — Correlations between S2 cell Dscam1 isoform frequencies from this experiment and from Sun et al [2] . Correlations between Sun et al [2] and A | S2 E. coli 12 hour exposure; B | S2 Control 12 hour exposure; C | S2 E. coli 18 hour exposure; D | S2 Control 18 hour exposure. Dscam1 isoform frequences from all four S2 treatments in this experiment correlated highly with isoform frequencies of the S2 cell isoform frequencies in Sun et al [2]: R-square values for the correlations and the corresponding p-values are in the top left corner of each scatterplot. (TIFF) [file pone.0108660.s004.tiff]
